# L’hidradénome papillifère

**DOI:** 10.11604/pamj.2017.26.196.11196

**Published:** 2017-04-04

**Authors:** Hanane Raiteb, Jaouad Kouach

**Affiliations:** 1 Service de Gynéco-Obstétrique, Hôpital Militaire d’Instruction Mohammed V, Rabat, Maroc; 2 Faculté de Médecine et de Pharmacie, Université Mohammed V Souissi, Rabat, Maroc

**Keywords:** Hidradénome papillifère, kyste, vulve, Hidradenoma papilliferum, cyst, vulva

## Image en médecine

Une primipare âgée de 25 ans consultait pour une lésion kystique du sillon interlabilial indolore mesurant 1,5 cm de couleur bleutée (A). L’exérèse est faite sous anesthésie locale. L’examen histologique montrait des projections papillaires vêtues par un revêtement apocrine (B) affirmant le diagnostic d’hidradénome papillifère (HP). C’est une tumeur annexielle bénigne rare, mais elle reste la plus fréquente des tumeurs glandulaires vulvaires (60 %) observée chez les femmes entre 20 et 90 ans, jamais avant la puberté. Il s’agit le plus souvent d’une lésion unique asymptomatique, de couleur muqueuse normale, bleutée ou bien rouge, de localisation vulvaire dans le sillon interlabial ou zones adjacentes au sillon. Sur le plan histologique c'est une tumeur kystique encapsulée, située dans le derme profond, sans connexion avec l'épiderme. Cette tumeur est remplie de villosités conjonctives, et la lumière est tapissée de deux assises cellulaires, une assise sécrétrice et une assise de petites cellules cuboïdes à noyaux très basophiles (cellules myoépithéliales). Il constitue probablement une prolifération adénomateuse des glandes ano-génitales de type mammaire. La simple exérèse est curative.

**Figure 1:**
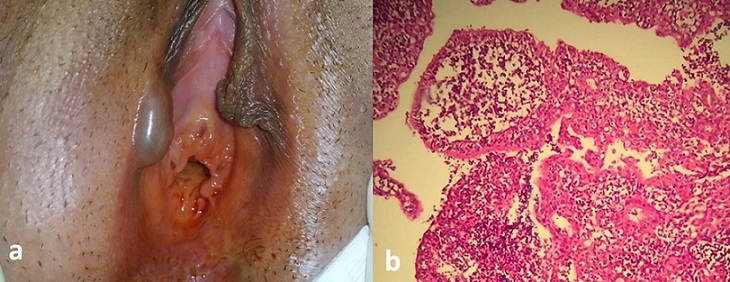
hidradénome papillifère A) aspact clinique de kyste du sillon interlabial; B) HE Gx10 tumeur annexielle bénigne faite de projections papillaires vêtus par un revêtement apocrine

